# A Highly Accurate, Polynomial-Based Digital Temperature Compensation for Piezoresistive Pressure Sensor in 180 nm CMOS Technology

**DOI:** 10.3390/s20185256

**Published:** 2020-09-14

**Authors:** Imran Ali, Muhammad Asif, Khuram Shehzad, Muhammad Riaz Ur Rehman, Dong Gyu Kim, Behnam Samadpoor Rikan, YoungGun Pu, Sang Sun Yoo, Kang-Yoon Lee

**Affiliations:** College of Information and Communication Engineering, Sungkyunkwan University (SKKU), Suwon 16419, Korea; imran.ali@skku.edu (I.A.); m.asif@skku.edu (M.A.); khuram1698@skku.edu (K.S.); riaz@skku.edu (M.R.U.R.); rlarlarbrb@skku.edu (D.G.K.); behnam@skku.edu (B.S.R.); hara1015@skku.edu (Y.P.); rapter@kaist.ac.kr (S.S.Y.)

**Keywords:** temperature compensation, digital controller, piezoresistive, pressure sensor, negative temperature coefficient, ACE-Q100, CMOS

## Abstract

Recently, piezoresistive-type (PRT) pressure sensors have been gaining attention in variety of applications due to their simplicity, low cost, miniature size and ruggedness. The electrical behavior of a pressure sensor is highly dependent on the temperature gradient which seriously degrades its reliability and reduces measurement accuracy. In this paper, polynomial-based adaptive digital temperature compensation is presented for automotive piezoresistive pressure sensor applications. The non-linear temperature dependency of a pressure sensor is accurately compensated for by incorporating opposite characteristics of the pressure sensor as a function of temperature. The compensation polynomial is fully implemented in a digital system and a scaling technique is introduced to enhance its accuracy. The resource sharing technique is adopted for minimizing controller area and power consumption. The negative temperature coefficient (NTC) instead of proportional to absolute temperature (PTAT) or complementary to absolute temperature (CTAT) is used as the temperature-sensing element since it offers the best temperature characteristics for grade 0 ambient temperature operating range according to the automotive electronics council (AEC) test qualification ACE-Q100. The shared structure approach uses an existing analog signal conditioning path, composed of a programmable gain amplifier (PGA) and an analog-to-digital converter (ADC). For improving the accuracy over wide range of temperature, a high-resolution sigma-delta ADC is integrated. The measured temperature compensation accuracy is within ±0.068% with full scale when temperature varies from −40 °C to 150 °C according to ACE-Q100. It takes 37 µs to compute the temperature compensation with a clock frequency of 10 MHz. The proposed technique is integrated in an automotive pressure sensor signal conditioning chip using a 180 nm complementary metal–oxide–semiconductor (CMOS) process.

## 1. Introduction

**Figure 1:**
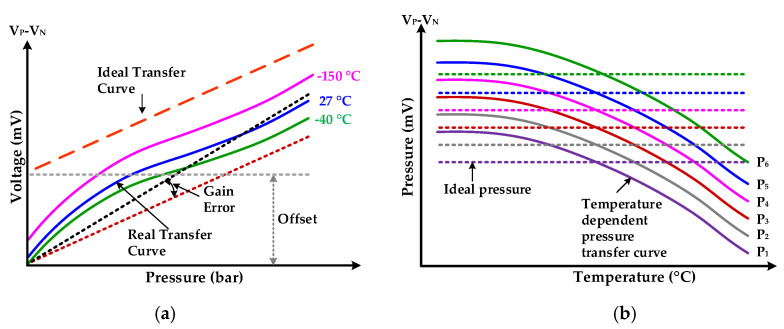
Piezoresistive-type (PRT) sensor transfer characteristics: (**a**) PRT input-output characteristics; (**b**) temperature-dependent pressure transfer curves at different constant pressures.

Presently, research on pressure sensors and transducers has been gaining significant attention. These sensors are being adapted widely in variety of applications such as the automotive industry, biomedical systems, petrochemicals, energy and electric power systems, aerospace, process control and humidity sensing systems [1,2,3,4]. The silicon piezoresistive-type (PRT) pressure sensor is widely used due to its simplicity, low cost, small size and robustness. In diverse harsh environment applications, the temperature increases up to 150 °C and consistency in sensor accuracy and performance are expected. For automotive applications, according to AEC-Q100 grade 0, the ambient temperature range is −40 °C to 150 °C [5]. The PRT sensor exhibits non-linear temperature dependency and its output voltage is influenced by temperature significantly. This complex temperature-dependent nature adversely affects the accuracy, reliability, precision and performance of piezoresistive sensors. The PRT sensor input-out and temperature dependent characteristics are elaborated in Figure 1 [6,7]. Other than non-linear behavior, gain and offset errors, depicted in Figure 1a, the PRT sensor output is highly dependent on temperature. At constant input pressure, the PRT sensor output voltage has complex relationship under dynamic temperature environment as shown in Figure 1b. Therefore, real time temperature compensation is mandatory for accurate and reliable measurement results of a sensor. In the harsh automotive environment, the temperature dependent pressure sensor variation must be encountered for accurate and reliable operation. The main drawback of current piezoresistive pressure sensors is the drop of output voltage with increase in the operating temperature which severely reduces the measurement accuracy. With the rise in temperature, the crystalline silicon electrical resistance increases and its piezoresistive coefficient decreases. The PRT sensor temperature sensitivity consists of temperature coefficient sensitivity (TCS) and temperature coefficient offset (TCO) [8]. The negative temperature coefficient of the piezoresistive coefficient is the main cause of TCS. The residual stress on packaging or membrane effects and mismatch values of resistors affects TCO. In the past, different techniques have been introduced to compensate for temperature variations in pressure sensors. Passive and active efforts were made to overcome undesired temperature effect on pressure output. In passive methods, additional resistors are utilized for temperature compensation in half bridge or full bridge arrangements within the sensor instead of signal conditioning circuit. Typically, the TCS and TCO are canceled by utilizing temperature-dependent series and trimmed parallel resistors. In active temperature compensation methodologies, additional temperature sensor is incorporated inside sensor chip. The TCS and TCO are compensated for by additional value from temperature sensor [8]. Based on the implementation method, these are categorized into hardware, software and hybrid approaches [9].

The passive compensation techniques were also adopted to eliminate pressure sensor output voltage drop with the increase in temperature [8,10,11]. For a piezoresistive pressure sensor, a built-in passive temperature compensation technique is introduced in [9]. An extra polysilicon resistor with negative temperature coefficient of resistivity (TCR) is employed inside a sensor-fabricated patch instead of the calibration process. In [11], a similar passive resistor temperature compensation method is presented in which the system parameters are manipulated by using differential equations. These passive techniques reduce TCS but TCO is not eliminated.

In the literature, several active methods using temperature element such as proportional to absolute temperature (PTAT), an analog-to-digital converter (ADC) and lookup tables are proposed [7,12,13]. A signal-conditioning integrated circuit (IC) is presented for piezoresistive pressure sensor in [7,13] in which temperature compensation incorporates on-chip PTAT used as the temperature element. The flash ADC converts the temperature analog signal to digital which is then used to pick a compensation factor from a look-up table. A similar approach is adopted in analog front end IC for an automotive capacitive pressure sensor [12]. A band gap reference (BGR) is used as temperature-sensing element and lookup approach is introduced for compensating temperature effect in pressure value. Such approaches are not accurate due to low-resolution ADC and the lookup table inside IC is integrated based on simulation results which causes errors due to PVT variation after fabrication.

Several software-based techniques using either conventional mathematical computation or artificial neural network (ANN) algorithms have been investigated to compensate for temperature effect on pressure sensor accuracy [14,15,16,17,18]. In [14], machine learning part is implemented on LabVIEW^®^ system for algorithm training and compensation part is designed on a microcontroller for a piezoresistive pressure sensor. The algorithm is trained first on the software system and then trained parameters are loaded into the microcontroller for real-time temperature compensation. However, the temperature compensation is valid only for the temperature range of −40 °C to 85 °C. Two techniques for capacitor pressure sensor modeling are reported in [15,16] which are based on a functional link ANN and back propagation neural network, respectively. These modeling based temperature compensation exhibits better accuracy of 1% FS only in a temperature range of −20 °C to 70 °C. An intelligent scheme using back propagation is proposed in [16] which achieves approximately 98% error reduction when applied to pressure ranges from 0 bar to 1 bar and temperature ranges from 25 °C to 80 °C. A feed-forward neural network is implemented on the CMOS analog application specific integrated circuit (ASIC) [17] for temperature compensation of a piezoresistive pressure sensor. With the presented technique, the error was reduced to 0.1% for compensated sensor in the temperature range of 0 °C to 70 °C. However, ANN-based proposed approaches are complex, requiring machine learning systems with large data set and exhibits low accuracy. Due to large computation and memory size, these methodologies are not appropriate for on-chip integration. These approaches do not clarify in neural networks the configuration and performance. A hybrid approach consisting of hardware and software for temperature compensation in pressure sensors is reported in [18]. It used a small processor as hardware compensation and a cubic B-spline based curve fitting algorithm in software. It is an off-chip compensation which results in an increased complexity. It is also unstable for batch compensation.

In this paper, a polynomial-based highly accurate temperature compensation technique is introduced. The compensation polynomial of a PRT pressure senor is proposed which is implemented in fully digital fashion. The negative temperature coefficient (NTC) sensor is used as a temperature-sensing element and high-resolution sigma-delta ADC (SD-ADC) is integrated. The NTC sensor, connected in Wheatstone bridge configuration has very high sensitivity of −3% to −6% per °C and it demonstrates comparatively very steep resistance-temperature slope and typically suitable for −55 °C to 200 °C temperature range. For automotive applications, according to AEC-Q100 grade 0, as shown in Table 1, it exhibits high accuracy for the ambient temperature range of −40 °C to 150 °C.

The rest of the paper is organized as follows: Section 2 presents architecture of pressure and temperature sensor interface chip top architecture. The detailed design of the proposed temperature compensation technique is described in Section 3. The temperature compensation digital controller is discussed in Section 4. The SD-ADC design is included in Section 5. Section 6 describes a programmable gain amplifier (PGA) design. Section 7 describes the experimental results and analysis. Lastly, the paper is concluded in Section 8.

## 2. Proposed Pressure Sensor Interface Architecture with Temperature Compensation

**Figure 2:**
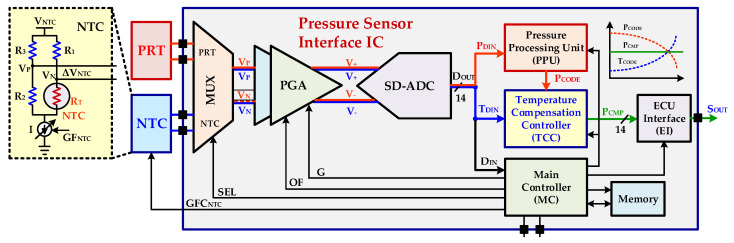
Automotive PRT pressure and temperature sensors signal interface integrated circuit (IC) with proposed polynomial based digital temperature compensation.

Typical signal conditioning integrated circuits nowadays usually perform analog and digital processing for improving automotive PRT sensor linearity, offset and gain errors [7,19]. Figure 2 shows the block diagram for a proposed PRT sensor interface IC with the presented polynomial-based digital temperature compensation. For automotive PRT pressure sensor, the highly reliable and accurate digital temperature compensation is composed of an off-chip NTC sensor, analog multiplexer (MUX), PGA, SD-ADC and polynomial-based configurable digital temperature-compensation controller (TCC). The proposed design may be integrated in any pressure sensor exhibiting this architecture with additional NTC sensor, MUX and TCC. The shared structure is introduced for pressure and temperature information processing. The main controller (MC) selects one of the sensor path for taking current pressure or temperature information from MUX. The sensor signal is amplified by PGA for increasing its range to a proper voltage level. The amplified signal is then fed to SD-ADC for digital conversion. In the proposed architecture, PGA and SD-ADC are shared for both PRT and NTC sensors and thus reduce cost and power consumption significantly. The pressure processing for non-linearities is performed in a pressure processing unit (PPU) while the real-time temperature compensation is achieved in the proposed polynomial-based TCC. The final pressure code with temperature compensation is delivered to an electronic control unit (ECU) interface (EI). The EI is either a digital to analog (DAC) converter with driving buffer amplifier (DA) [7] or it is a digital serial interface such as single edge nibble transmission (SENT) [19]. In the proposed pressure sensor interface chip, SENT is incorporated for its interface with the ECU and the final output signal SOUT is an asynchronous digital signal. Since, the pressure and temperature signals have very low frequencies of a few kHz, therefore, a low speed, high resolution SD-ADC is used for precise digitization of analog signals. The digital processing is more robust and reliable compared to analog processing [20,21]. Also, digital compensation processing is much easier and simpler than in analog techniques. Therefore, the digital temperature compensation and processing approach is adapted in the proposed system.

## 3. Proposed Temperature Compensation

**Figure 3:**
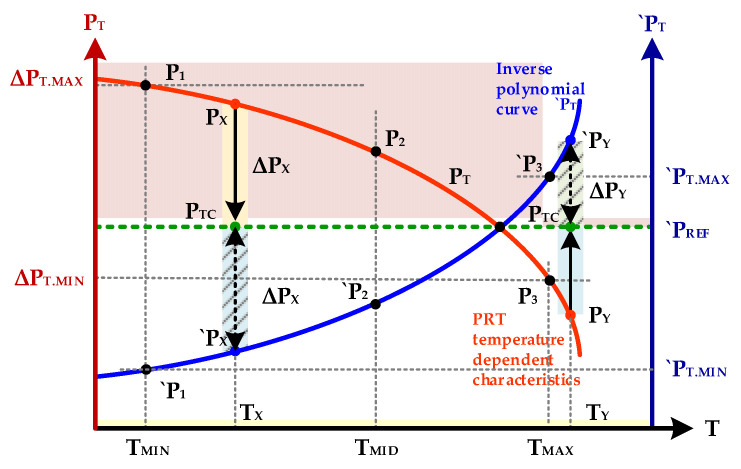
Concept of pressure and its compensation polynomials.

The main drawback of current piezoresistive pressure sensors is the drop of output voltage with the increase in the operating temperature which severely reduces the measurement accuracy. Ensuring the accurate operation of the sensor with temperature variation is critical to satisfy the temperature characteristics of the AEC-Q100 [5]. A novel polynomial-based technique is introduced in a digital way to compensate temperature variations. The concept of temperature-dependent PRT characteristics and its compensation polynomial is depicted in Figure 3. The output voltage of the pressure sensor is not a linear function of temperature and the input-output of a pressure sensor have a complex polynomial relationship. Thus the opposite of the polynomial is an ideal solution to compensate temperature variation accurately with zero error for the full range of temperature.

The NTC thermistor is connected in a Wheatstone bridge configuration to detect output voltage variation ΔV_NTC_ as a function of temperature. The NTC gauge factor calibration keeps the NTC output voltage to a certain range. When the NTC sensor is connected to PGA through MUX, after gain, and offset calibration of PGA for NTC sensor signal, the temperature information is converted to digital by a 14-bit SD-ADC. The TCC is a finite state machine (FSM) based configurable digital controller which integrates compensation polynomial characteristics of pressure sensor as function of temperature. Based on the characteristics of a PRT sensor, the coefficients of compensation polynomial may be very small with fractional parts. The scaling technique is introduced to eliminate errors due to fractional part which results in more accurate temperature compensation.

**Figure 4:**
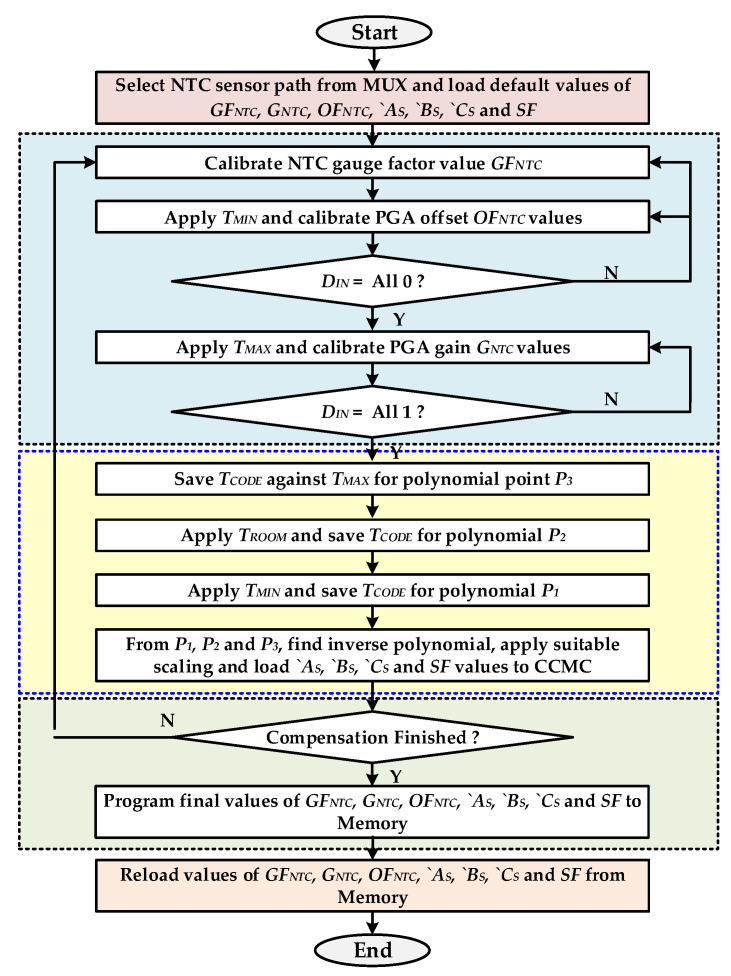
Temperature compensation parameter preparation flow chart.

Figure 4 shows the flowchart for preparing temperature compensation parameters. The different parameters like the NTC sensor gauge factor, PGA gain and offset for NTC sensor, and compensation polynomial coefficients are computed before starting temperature compensation. The proposed temperature compensation parameters are achieved with the following steps:

1 The MC selects the NTC sensor path from MUX and applies default values from memory for the gauge factor GF_NTC_, PGA offset OF_NTC_ and gain G_NTC_ for NTC sensor.
2 The NTC gauge factor is set to its central value and the PGA offset and gain is determined automatically by the MC for the NTC sensor. For offset cancelation, the minimum temperature is applied and the PGA is tuned to make ADC output nearest to zero. Then the highest temperature is applied and PGA gain is tuned to get the highest possible value of ADC output.
3 To find out the temperature compensation parameters, fixed pressure is applied at the PRT sensor input. The temperature of chip is changed from minimum *T_MIN_* to maximum *T_MAX_*. Due to non-linear temperature-dependent PRT characteristics, the sensor output voltages decreases when temperature is swept from *T_MIN_* to *T_MAX_* with even fix pressure at its input. Three values of the pressure code from ADC output in digital format are achieved when the temperature values are −40 °C (minimum), 25 °C (mid) and 150 °C (maximum), respectively. These values give the three points *P*_1_, *P*_2_ and *P*_3_ for the complex temperature-dependent pressure characteristics of PRT sensor as shown in Figure 4. The second-degree polynomial representing this relationship is give as in Equation (1):
  (1) $$P_{T}=aT^{2}+bT+c$$
  where *a*, *b* and *c* are the coefficients of polynomial, *T* is temperature and *P_T_* is the temperature-dependent pressure value. This polynomial is valid if temperature is swept at different constant input pressure.

4 Compensation characteristic is computed from the temperature-dependent pressure characteristics with three polynomial points *`P*_1_,*`P*_2_ and *`P*_3_ and is given in Equation (2) as follows:

(2) $$`P_{T}=AT^{2}+BT+C$$

5 Since the coefficients *A*, *B* and *C* may have very small values depending on the curve shape for different sensors, so for digital implement with enhanced accuracy of the polynomial, the scaling technique is introduced. Both sides of the equation are multiplied by a suitable number 2*^SF^* so that the smallest coefficient has significant integer value, where *SF* is scaling factor.
  (3) $$2^{SF}.`P(T)=2^{SF}.(AT^{2}+BT+C)$$
  (4) $$`P_{S}=2^{SF}AT^{2}+2^{SF}BT+2^{SF}C$$
  (5) $$`P_{S}=A_{S}T^{2}+B_{S}T+C_{S}$$

The final compensation of the polynomial in Equation (5) is designed and implemented in TCC. During normal operation after temperature compensation and pressure calibration, the compensating value is determined by downscaling the result of Equation (5) by the same factor of 2*^SF^* as follows in Equation (6):

(6) $$`P=\frac{`P_{S}}{2^{SF}}$$

Since, in downscaling step, the division is involved. The scaling value is selected in the form of the power of 2. This technique eliminates the necessity for a binary divider and downscaling is accomplished by hardware friendly right shift operation.

6 The memory is programmed with compensated parameters of gauge facture *GF_NTC_*, PGA offset *OF_NTC_*, PGA gain *G_NTC_* and compensation polynomial coefficients *A_S_*, *B_S_*, *C* and scaling factor *SF*. After reset, these parameters are automatically loaded from memory and are used during temperature compensation.

During normal operation, when the IC is reset or powered on, the polynomial and temperature-compensation parameters are loaded from memory to TCC and MC. Since, the NTC and PRT share the same PGA and SD-ADC, therefore at a time one path is selected by MC. The temperature change rate is not so high and the temperature path selection is less frequent compared to pressure sensing duration and most of the time, the PRT path is selected. The final temperature-compensated pressure code *P_CMP_* is given as follows in Equation (7):

(7) $$P_{CMP}=P_{CODE}\pm \Delta P$$

where, *P_CODE_* is uncompensated pressure code after PPU processing and Δ*P* is pressure variation due to temperature which is compensated for by the proposed design. During normal chip operation, if some pressure having digital code of *P_X_* at temperature *T_X_* is applied then it means due to temperature variation the sensor output is more than the real value. This addition pressure variation need to be subtracted from *P_X_* to obtain *P_CMP_* as shown in Figure 3 and is given as follows in Equation (8):

(8) $$P_{CMP}=P_{X}-\Delta P$$

In this case, when *`P_T_* is less than a reference value *`P_REF_*, the Δ*P* is the difference of *`P_REF_* and *`P_T_* and is described in Equation (9) as follows:

(9) $$\Delta P=`P_{REF}-`P_{X}$$

Hence, the final compensated value in this case is computed as follows in Equation (10):

(10) $$P_{CMP}=P_{X}-(`P_{REF}-`P_{X})$$

Similarly, if *`P_T_* value at current temperature *T_Y_* is greater than *`P_REF_*, then it shows the pressure code is less than the actual value. As is clear from Figure 3, in this case pressure variation of Δ*P_Y_* is mandatory to add in *P_Y_* to obtain temperature-compensated *P_CMP_* as explained in Equation (11):

(11) $$P_{CMP}=P_{Y}+(`P_{Y}-`P_{REF})$$

Finally, if the *`P_T_* is equal to *`P_REF_* then it means, there pressure code represents the actual value and no compensation is needed as clear from conceptual diagram elaborated in Figure 3. In general, all three *P_CMP_* computation scenarios are summarized as follows in Equation (12):

(12) $$P_{CMP}=\left\{ \begin{array}{ll} P_{CODE}-(`P_{REF}-`P_{T}) & if\;`P_{T}<`P_{REF}\;\\ P_{CODE} & if\;`P_{T}=`P_{REF}\\ P_{CODE}+(`P_{T}-`P_{REF}) & if\;`P_{T}>`P_{REF} \end{array}\right.$$

The reference *`P_REF_* is configurable and its position is adjustable during compensation polynomial design.

## 4. Temperature-Compensation Controller (TCC)

**Figure 5:**
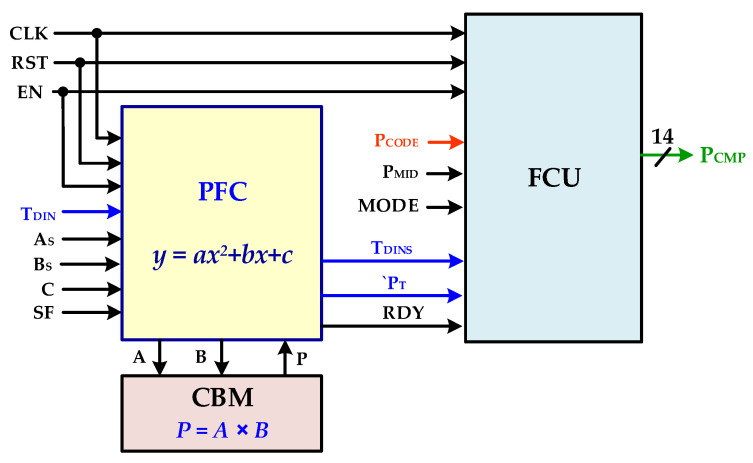
Proposed temperature-compensation controller (TCC) architecture.

The main core of the proposed polynomial-based temperature compensation is designed as a configurable digital controller. Since, recent research focuses on the digital solutions rather than analog circuits due to simplicity, scalability, noise immunity along with less power consumption and reduced area requirement, and therefore the proposed compensation polynomial is designed as fully digital circuit. Figure 5 shows the block level architecture of the TCC which is mainly composed of a polynomial FSM controller (PFC), final compensation unit (FCU) and combinational binary multiplier (CBM). When enabled, the PFC computes the polynomial for configured parameters based on the current temperature value. Resource sharing is adopted and a single CBM block is reused for several timely managed multiplication operations to reduce occupied area and power consumption. The configurable TCC architecture is very flexible and easily scalable for computing any higher degree polynomial computation at the cost of additional clock cycles. The PFC computes polynomial value for current input temperature code *T_DIN_* when the NTC sensor path is selected. The *A_S_*, *B_S_* and *C* are configurable polynomial coefficients. The coefficients *A*_S_ and *B*_S_ are scaled by factor of 2*^SF^*. The PFC is designed with a finite state machine control unit and related datapath which mainly computes polynomial in several clock cycles. Single-cycle polynomial computation architecture is also possible at the cost of more hardware for parallel computing. In current design, sequential architecture is adopted with minimum possible hardware utilization because the temperature variation is not abrupt and polynomial evaluation is possible in a very short time interval.

**Figure 6:**
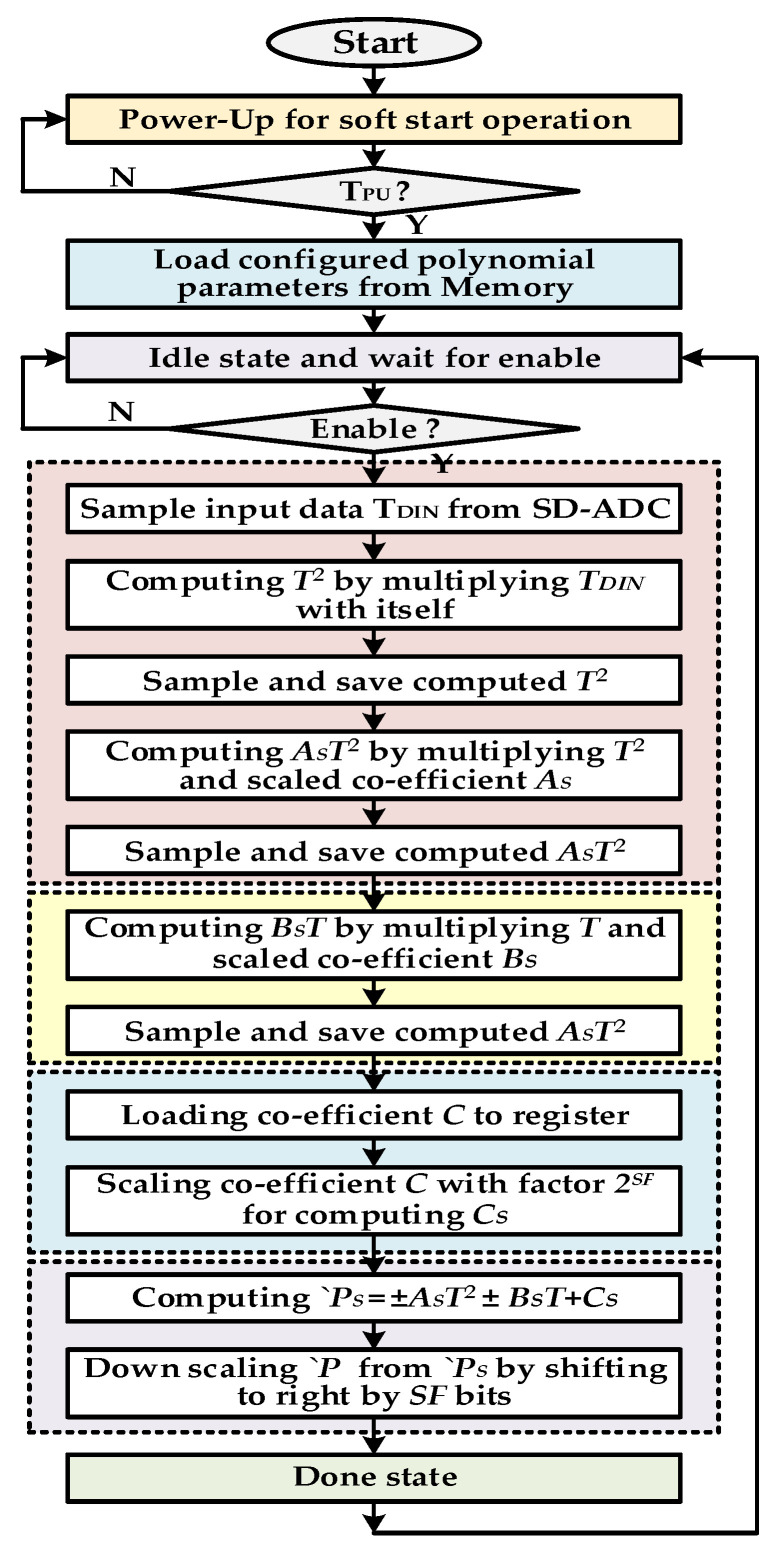
Polynomial finite state machine (FSM) controller flow chart.

The PFC flow diagram is depicted in Figure 6. The controller remains in power-up state for configured duration *T_PU_* after turning on or being reset. This allows the other blocks of the chip to be settled down after soft start and also prohibits TCC contributing to the inrush current. The polynomial parameters such as *A_S_*, *B_S_*, *C* and scaling factor *SF* are saved in the memory after the compensation procedure and are loaded in TCC registers on power-up. When the signal conditioning chip is in normal operation then PFC waits for the enable signal from MC. The MC first selects the NTC path and enable TCC. The current temperature digital code *T_DIN_* is sampled in internal register and polynomial manipulation starts. In the first phase, *AT^2^* is computed. For this first *T*^2^ is calculated by CBM and its result is saved; then in the second multiplication *A_S_* and *T*^2^ are multiplied and final *A_S_T*^2^ is saved in internal register. Then in next phase, *BsT* is computed and saved in a separate register. The coefficient *C* is unscaled to reduce its size unlike other scaled coefficients *A_S_* and *B_S_* and, therefore, it is scaled to factor 2*^SF^*. The final polynomial value *`P_S_* is calculated and down scaled to original value *`P* by just right sift operation. The scaling value is chosen as power of 2. This eliminates the requirement of the binary divider and division is achieved by a simple right shift operation. This technique reduces significant area and cost. When compensation value *`P* based on current temperature and parameters is ready, then FCU computes the final compensated code *P_CMP_* as according to Equation (12). During the pressure path, the current pressure digital value *P_DIN_* is processed by PPU and its value *P_CODE_* remains hold during NTC path. When *`P* is computed, then it is used for pressure compensation. If *T_DIN_* is less than *P_MID_*, it means pressure value is higher than the ideal value. In this case, *P_CODE_* needs to be reduced sufficiently so that it becomes equivalent to *P_MID_*. Similarly, *P_CODE_* needs to be increased by an amount if *T_DIN_* is greater than *P_MID_*. When *T_DIN_* code is same as *P_MID_* then *`P* will also be equal to *P_IMID_* which means the pressure code is already equal to *P_MID_* and does not need to be compensated for. In the proposed structure, compensation polynomial of both positive and negative slope is designed and selectable from the main controller.

## 5. Sigma-Delta Analog-to-Digital Converter

**Figure 7:**
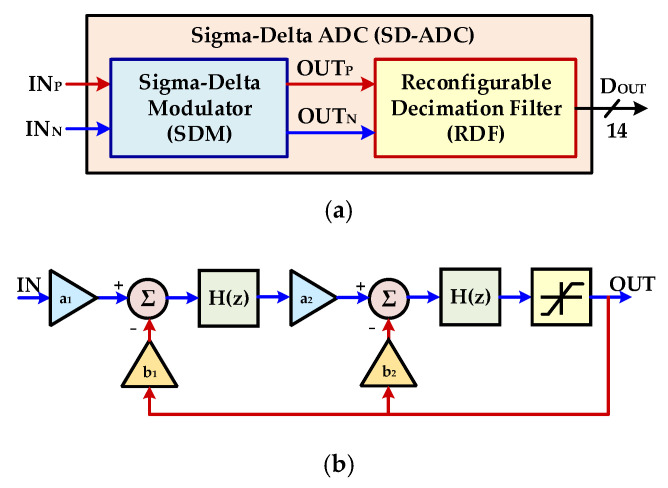

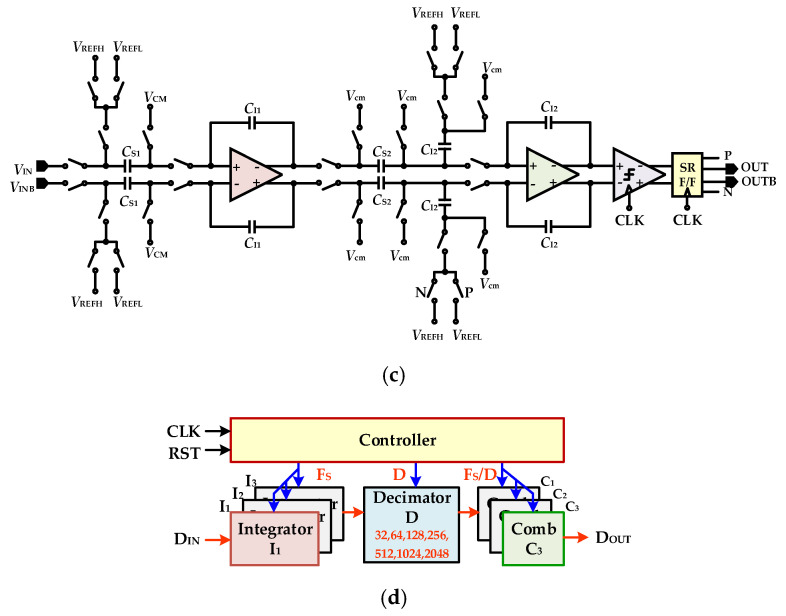
Sigma-Delta analog-to-digital converter (ADC): (**a**) block diagram with sigma-delta modulator (SDM) and reconfigurable decimation filter (RDF); (**b**) second order SD-ADC block diagram; (**c**) second order SD-ADC circuit diagram; (**d**) reconfigurable decimation filter (RDF) architecture.

The ADCs are implemented on system-on-chips (SoCs) so that converted digital data may be used for further digital processing. In the proposed architecture, the signal frequencies to the ADC are in the low range and precise digitized signals are required, which leads to the design of a low speed and high resolution ADC. In the previous designs, successive approximation register (SAR) ADC has been used for this purpose due to its low power consumption. This ADC structure is not suitable for high-accuracy measurements as it has a limitation in terms of resolution [22,23,24,25]. A reconfigurable second-order SD-ADC is designed for automotive PRT sensor. In the proposed temperature-compensation architecture, the existing ADC is shared for digitalizing the NTC temperature signal and, therefore, n additional converter is not required. In SD-ADC, different techniques at system and circuit levels have been implemented to address the design challenges. Figure 7a represents a simplified top-block diagram of the SD-ADC. The proposed ADC consists of a second-order sigma-delta modulator (SDM) and reconfigurable decimation filter (RDF). The chopper stabilization technique is used in each SDM integrator stage to reduce the influence of low-frequency noise and offset error. The placement of each cell is also optimized to obtain the required specification of the SD-ADC. A non-overlap clock generator circuit to overcome delay differences is also implemented in the proposed design. In order to ensure better stability performance and low area, a second-order discrete-time (DT) SDM with a cascaded integrator feedback (CIFB) structure is realized. The block diagram of the second order SDM is shown in Figure 7b. The modulator consists of two switched capacitor integrators whose output is fed back to the integrators with the coefficients *b_1_* and *b_2_*. The values of these coefficients have been derived and fixed through Simulink^®^ modeling which is done in MATLAB^®^. Figure 7c shows the schematic level implementation of the second order SDM. The coefficients *a*1 and *a_2_* are implemented as the ratio of two capacitances *C_S1_/C_I1_* and *C_S2_/C_I2_*, respectively. In the modulator, top and bottom reference voltages, *V_REFH_* and *V_REFL_*, define the feedback coefficients *b_1_* and *b_2_* with the optimized values of 0.25 and 1.0, respectively. A gain-boosted topology is employed for amplifier used in the integrator stages of SDM. The folded-cascode with P-type and N-type structures are used in gain-boosted structure. Moreover, the chopper stabilization technique is used to minimize the influence of *1/f* noise at lower frequencies. Moreover, a common mode feedback (CMFB) is applied to all amplifiers to keep the biasing level. The structure uses a single bit quantizer, consisting of a dynamic type comparator followed by two set/reset (SR) latch cells, which determine the outputs of the comparator. For small area and low power, CIC filter structure is used as a decimation filter for SDM [26]. Figure 7d shows that a digital controller is used along with CIC filter to make it reconfigurable and hence it is used for different date rates and input signal bandwidth. The decimation factor is configurable among 32, 64, 128, 256, 512, 1024 and 2048 depending upon the required output data rate and input signal.

## 6. Programmable Gain Amplifier with Offset Compensation and Single to Differential Circuits

**Figure 8:**
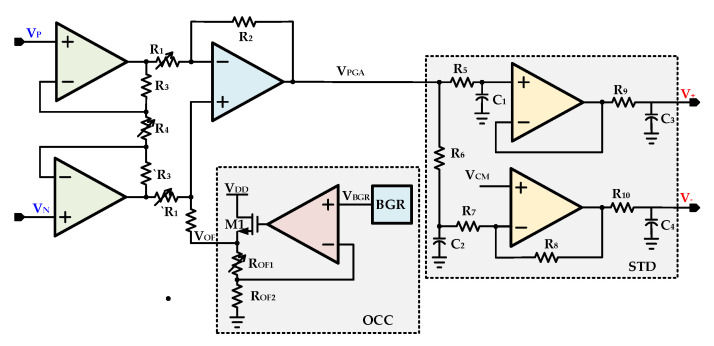
Programmable gain amplifier (PGA) with offset compensation (OCC) and single to differential (STD) circuits.

The programmable gain amplifier with offset compensation circuit (OCC) [7] and single to differential (STD) is shown in Figure 8. The PGA is designed with three amplifiers and an additional offset compensation block. To meet the performance requirements, such as high immunity towards noise and a large signal-to-noise ratio, a differential signal is needed to drive the ADC. A single to differential amplifier is designed with two amplifiers to convert a single-ended PGA output signal *V_PGA_* to a differential signal pairs *V_+_* and *V_-_* as shown in Figure 8. The PGA amplifies the difference between input signals VP and VN by gain of APGA as described in Equation (13) as follows:

(13) $$V_{PGA}-V_{OF}=A_{PGA}(V_{P}-V_{N})$$

The PGA gain, *A_PGA_* is depended and controlled by ratio of resistors R_1_ and R_4_ and is give as follows in Equation (14).

(14) $$V_{PGA}=V_{OF}-\Delta RI(1+\frac{2R_{3}}{R_{4}})\frac{R_{2}}{R_{1}}$$

where, *I* is current passing through NTC or PRT sensor and *V_OF_* is OCC offset voltage value. With the designed resistor values, the PGA gain is controllable from 4.88 V/V to 25.83 V/V with step size of 0.655 V/V. The designed bandwidth is 2 MHz at maximum PGA gain of 25.83 dB. In the pressure sensor interface IC, the PGA facilitates the digital conversion process for SD-ADC by the amplifying voltage signal obtained from PRT or NTC sensors and enhances resolution. The output voltage for each sensor differs slightly depending on the pressure and temperature. This voltage difference is compensated for by controlling the gain. The offset voltage *V_OF_* is applied from OCC. For generating constant offset voltage for PGA, the compensation circuit in low drop-out structure is used. The *V_OF_* depends on resistors ratio and is obtained by Equation (15) as follows:

(15) $$V_{OF}=(1+\frac{R_{OF1}}{R_{OF2}})V_{BGR}$$

where *V_BGR_* is BGR output voltage. The PGA should not exceed the input range of the ADC at the pressure range. Therefore, PGA gain and offset are controlled by resistors *R_OF1_*, *R_1_*, and *R_4_* from MC These resistors are implemented as resistor backs which are controllable form MC. The final calibrated resistors codes are stored in memory by the MC digital controller. During normal operation, the calibrated resistor values are fetched from memory and are applied before reading signal values from NTC or PRT sensors.

## 7. Experimental Results

**Figure 9:**
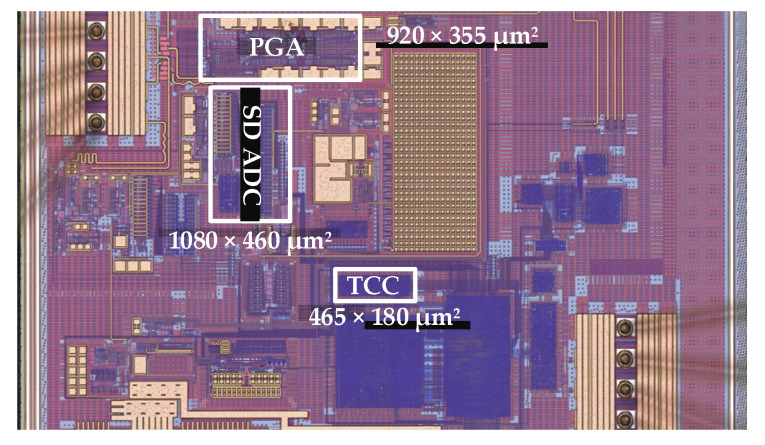
Chip microphotograph.

**Figure 10:**
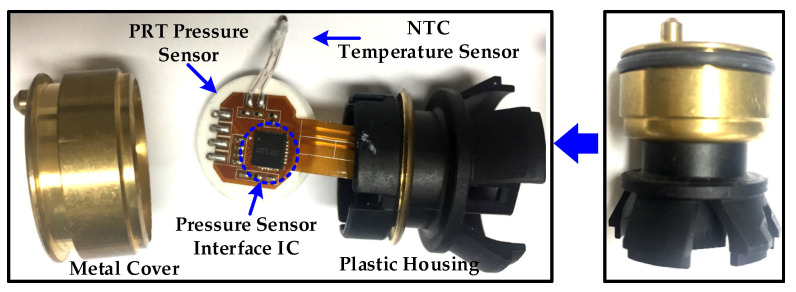
PRT pressure sensor module with PRT and negative temperature coefficient (NTC) sensors and proposed temperature compensation.

The proposed polynomial-based digital temperature compensation is integrated in piezoresistive type pressure sensor signal conditioning IC for automotive applications. The design is fabricated with a 1P6M 180 nm CMOS process. The chip microphotograph is depicted in Figure 9 in which TCC, SD-ADC and PGA are highlighted. The existing analog front end (AFE) and ADC used for the pressure path are shared and only with the integration of TCC, the proposed technique is implemented. The TCC is fully synthesizable and occupies only 465 × 180 µm^2^ area. The performance of the temperature-compensation controller is summarized in Table 2. The fully scalable and configurable TCC architecture requires only 1386 K logic gates for its full implementation and consumes only 1375 µW for its full operation. Figure 10 shows the pressure sensor module with the integrated chip including the proposed digital compensation. Inside metal cover, PRT, NTC and pressure sensor interface chip are stuffed on a flexible printed circuit board (PCB). The NTC sensor is connected in Wheatstone bridge configuration. The NTC sensor has very high sensitivity of −3% to −6% per °C compared to PTAT or a resistance temperature detector (RTD). It demonstrates comparatively very steep resistance-temperature slope and typically suitable for the −55 °C to 200 °C temperature range. In the pressure sensor interface IC, the NTC sensor is used for temperature information and temperature calibration.

The performance comparisons of the proposed temperature compensation with the exiting methods are summarized in Table 3. Most of the prior works adopted PTAT and BGR as the temperature sensor element. Their sensitivity is very low compared to NTC. The NTC exhibits very high sensitivity in the required temperature range according to AEC-Q100 grade 0. In prior works, the lookup table (LUT) which is based on simulation analysis is used which results in poor performance. Furthermore, low-resolution ADC in previous works also limits the accuracy. In the proposed work, very high-sensitivity NTC temperature element and a 14-bit ADC are used for polynomial-based digital on the fly compensation.

The accuracy is the ratio of deviation of output *N_MEAS_* from ideal value *N_IDEAL_* to full-scale output value *N_FOS_*, and is given as follows in Equation (16):

(16) $$Accuracy=Min\left\{1-\frac{N_{MEAS}-N_{IDEAL}}{N_{FSO}}\right\}\times 100(\%)$$

After PGA gain and offset compensation, the minimum and maximum measured digital temperature codes are 68 and 16,285 when chip temperature is −40 °C and 150 °C, respectively, at fixed pressure. With this measured range, the full-scale value is 16,217. The maximum measured deviation of digital code from ideal value is 11. With these values, from Equation (16) the accuracy is 99.9321%. Hence, the output temperature compensation accuracy is within ±0.06783% with full scale.

**Figure 11:**
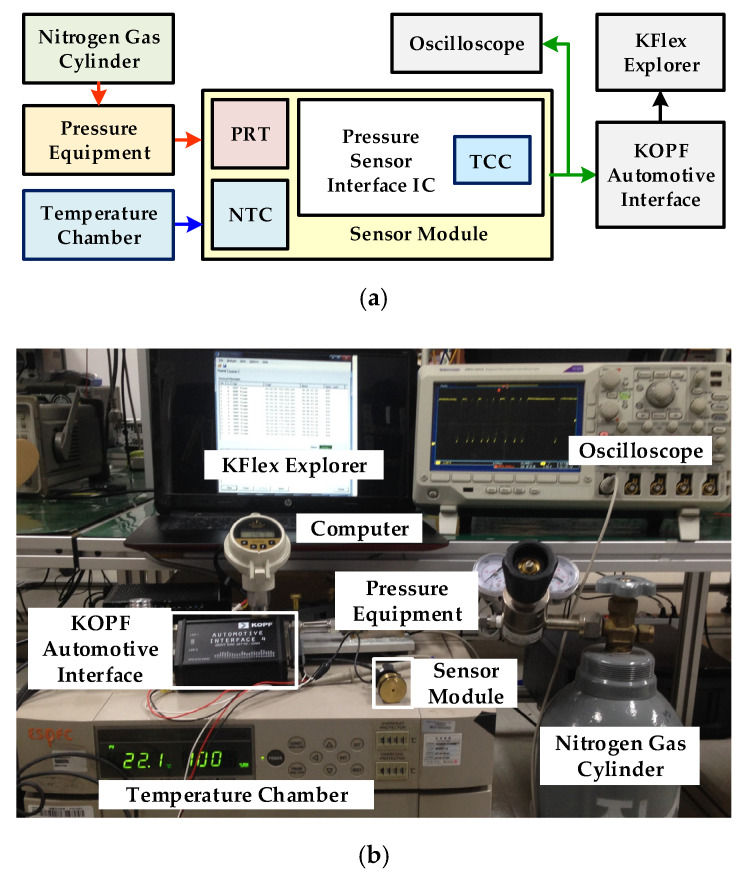
Experiment environment: (**a**) measurement block diagram; (**b**) experimental lab setup.

**Figure 12:**
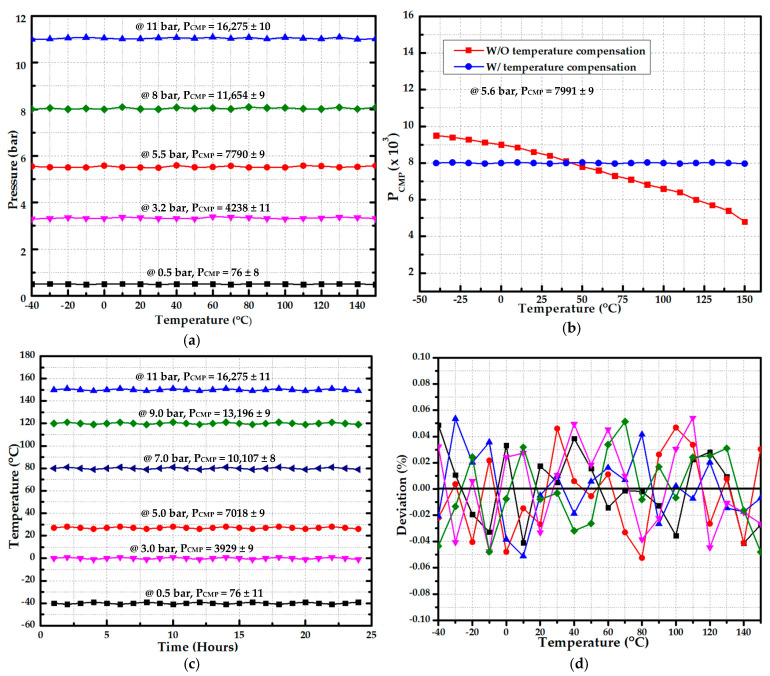
Temperature-compensation measurement results: (**a**) temperature compensation results at different input pressure; (**b**) with and without temperature compensation at 5.6 bar input pressure; (**c**) 24-h measurement at different fixed pressure and constant temperature; (**d**) percentage output deviation from ideal value at different pressures with full temperature range.

Figure 11 explains the experimental environment for measuring temperature-compensation performance. The pressure is applied from nitrogen gas cylinder and different temperature conditions are measured with temperature chamber. The digital serial output is received on a computer for analysis. Different temperature-compensation measurement results are depicted in Figure 12.

In Figure 12a, various fixed pressures are applied and the temperature is changed from –40 °C to 150 °C. The pressure range is 0.5 bar to 11.0 bar. The maximum deviation from ideal value is 11 which results in 0.06783% accuracy including ADC noise. The measurement with and without temperature compensation is depicted in Figure 12b in which fixed 5.6 bar pressure is applied. The ideal digital output is 7991 whereas the maximum deviation of ±9 is reported. The fixed pressure is applied at fixed temperature for 24 h and proposed temperature compensation performance is analyzed. The results are summarized in Figure 12c. As is clear from results, the digital output is almost constant with variation in four least significant bits. Figure 12d explains the percentage output deviation from ideal value for various fixed pressures while sweeping temperature. It also includes ADC noise with input DC value.

**Figure 13:**
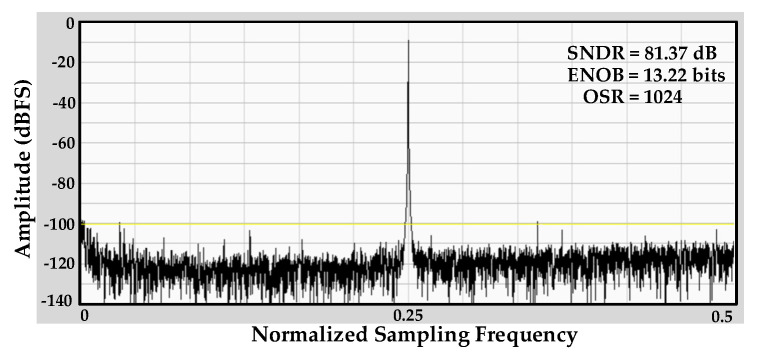
Measured sigma-delta analog-to-digital converter (SD-ADC) fast Fourier transform (FFT) spectrum.

Figure 13 shows the measured fast Fourier transform (FFT) spectrum of SD-ADC with an effective number of bits (ENOB) of 13.22 bits and signal-to-noise and distortion ratio (SNDR) level of 81.37 dB. The measurement is done with 0.61 kHz input signal frequency (*f_IN_*), input signal level of 300 mV and an OSR 1024 operating at a sampling frequency (*f_S_*) of 2.5 MHz.

**Figure 14:**
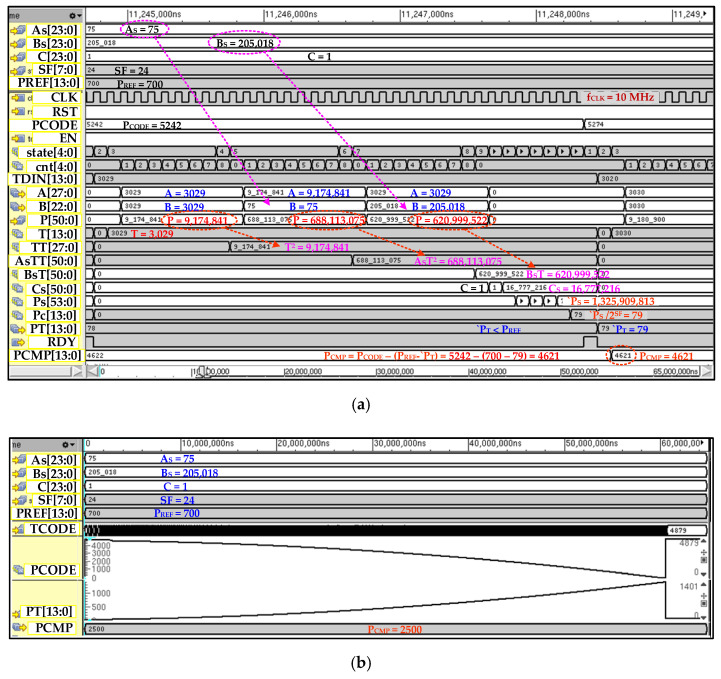
TCC simulation results: (**a**) polynomial computation and temperature compensation for single iteration; (**b**) full temperature sweep from −40 C to 150 C with constant input pressure.

The detailed post-place and route (P&R) simulation results with a NC-Verilog^®^ simulator of TCC are elaborated in Figure 14. The polynomial computation for a single input of temperature value is shown in Figure 14a. The polynomial coefficient parameters *A_S_*, *B_S_*, *C* and *SF* are configured to 75, 205,018, 1 and 24, respectively. The *P_REF_* and *P_CODE_* are 700 and 5242, respectively. In this simulation, the temperature *T_DIN_* is 3029 when it is enabled from main controller. After enabling, TCC takes 37 clock cycles to compute compensating value *`P_T_* of 79 which is adjusted to pressure code to compute compensated pressure *P_CMP_* of 4621. The full temperature-seep simulation results are shown in Figure 14b. It is clear from results that the *`P_T_* is perfect compensation curve of temperature-dependent pressure sensor characteristics represented as *P_CODE_*.

**Figure 15:**
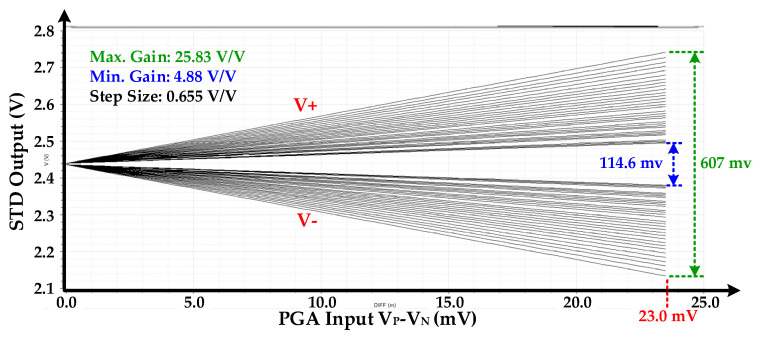
Programmable gain amplifier (PGA) simulation results at different gain including STD.

The PGA simulation results including STD for entire configurable gain range is depicted in Figure 15. The S2D differential output voltage ranges from 114.6 mV to 607 mV when PGA input voltage difference is 23.0 mV. The PGA gain is controllable from 4.88 V/V to 25.83 V/V with step size of 0.655 V/V. The gain is controlled from MC with G <4:0> signal. The configurable PGA gain is acceptable for the SD-ADC with 300 mV of peak-to-peak input.

## 8. Conclusions

A highly accurate, polynomial-based adaptive digital temperature compensation is presented for automotive piezoresistive pressure sensor applications. By integrating compensation characteristics of the pressure sensor as function of temperature, non-ideal temperature dependency of the pressure sensor is accurately compensated. The compensation polynomial is fully implemented in a digital form with a scaling technique introduced to enhance its accuracy. For area and power efficient design, a resource-sharing technique is adopted. The NTC instead of PTAT or CTAT is used as a temperature-sensing element as it offers the best temperature characteristics for ACE-Q100 grade 0 ambient temperature operating range. A high-resolution 14-bit SD-ADC is proposed for improving accuracy over a wide temperature range. When temperature varies from −40 °C to 150 °C according to ACE-Q100, temperature compensation accuracy reported is 99.93% and it is within ±0.068% at full scale. It takes 37 µs to compute the temperature compensation with 10 MHz of clock frequency. The proposed technique is integrated in an automotive pressure sensor signal conditioning IC using a 1P6M 180 nm CMOS process.

## Figures and Tables

**Table 1:** AEC-Q100 compliant operating temperature grades.

Grade	Ambient Operating Temperature Range
Grade 0	−40 °C to +150 °C
Grade 1	−40 °C to +125 °C
Grade 2	−40 °C to +105 °C
Grade 3	−40 °C to +85 °C
Grade 4	0 °C to +70 °C

**Table 2:** TCC performance summary.

Parameter	Value
CMOS process	180 nm
Occupied area	0.0837 mm^2^
Gate count	1.386 K
Supply voltage	1.8 V
Current consumption	764 nA
Power consumption	1.375 µW
Clock Frequency	10 MHz
Polynomial	2nd Order
Scalable	Yes
Configurable architecture	Yes

**Table 3:** Temperature-compensation performance comparison.

Parameter	[7]	[12]	[13]	[27]	This Work
CMOS process (µm)	0.35	0.35	0.18	-	0.18
System clock (MHz)	4	8.96	4	-	10
Power consumption (mW) ^1^	23.5	25	11.8–64.8	20	22.5
Pressure sensor type	PRT	Capacitive	PRT	SOS ^2^	PRT
Temperature range (°C)	−40–+150	−30–120	−40–+85	−20–+140	−40–+150
Temperature method	LUT ^3^	LUT ^3^	Digital	Software	PDTC ^4^
Temperature sensor	PTAT	BGR	PTAT	RTD ^5^	NTC
ADC type	Flash	Flash	Charge balancing	Sigma-Delta	Sigma-Delta
ADC resolution	4	4-bit	16	24	14
Deviation (%) FS ^6^	0.5	1.0	0.1	0.3	0.068

^1^ Chip current and supply voltage. ^2^ Silicon-on-Sapphire. ^3^ Lookup table-based temperature compensation. ^4^ Polynomial-based digital temperature compensation. ^5^ Resistance temperature detector. ^6^ Temperature compensation deviation from ideal value for full scale (FS).
